# Antimicrobial Resistance Patterns of Bacterial Isolates from Blood Culture among HIV/AIDS Patients at Felege Hiwot Referral Hospital, Northwest Ethiopia

**DOI:** 10.1155/2020/8893266

**Published:** 2020-10-19

**Authors:** Mohabaw Jemal, Teshiwal Deress, Teshome Belachew, Yesuf Adem

**Affiliations:** ^1^University of Gondar, College of Medicine and Health Sciences, School of Biomedical and Laboratory Sciences, Department of Medical Microbiology, Gondar, Ethiopia; ^2^University of Gondar, College of Medicine and Health Sciences, School of Biomedical and Laboratory Sciences, Unit of Quality Assurance and Laboratory Management, Gondar, Ethiopia; ^3^Bahir Dar University, College of Medicine and Health Sciences, School of Health Sciences, Department of Medical Laboratory Sciences, Unit of Medical Microbiology, Bahir Dar, Ethiopia

## Abstract

**Background:**

The emergence and spread of antimicrobial resistance in bacteria is recognized as a global public health problem. Bloodstream infection with antimicrobial-resistant bacteria in HIV/AIDS patients makes the problem more challenging. So, regular and periodic diagnosis and use of the appropriate antimicrobial susceptibility pattern determination is the only option for decreasing the prevalence and development of drug-resistant bacteria.

**Methods:**

An institution-based cross-sectional study was conducted among 384 HIV/AIDS patients. Sociodemographic data of patients were recorded using structured questionnaires. Blood cultures were collected with BACTEC aerobic blood culture bottles. A pair of samples was collected from each patient aseptically and incubated at 37°. If samples are positive for bacterial agents, they were subcultured to solid media such as blood agar plate, chocolate agar plate, and MacConkey agar plates. Identification was performed using colony characteristics and standard biochemical techniques. The antimicrobial susceptibility test was determined by the Kirby–Bauer disc diffusion method. Data entry and analysis were performed while using SPSS version 20. Descriptive statistics were performed to calculate frequencies.

**Results:**

Altogether, 384 patients were included, and 123 blood cultures were positive, so that the yield was thus 32%. About 46 (37.4%) of Gram-negative and 77 (62.6%) of Gram-positive bacterial species were identified. Among Gram-negative bacterial isolates, *K. pneumoniae* was the leading pathogen, 19 (41.3%), whereas *S. aureus*, 38 (49.4%), was predominant among Gram-positive isolates. In his study, the majority of Gram-positive isolates showed high level of resistance to penicillin, 72 (95.5%), tetracycline, 55 (71.4%), and cotrimoxazole, 45 (58.4%). About 28 (73.6%) of *S. aureus* isolates were also methicillin-resistant. Gram-negative bacterial isolates also showed a high resistance to ampicillin (91.3%), tetracycline (91.3%), and gentamicin (47.8%). Overall, about 78% of multidrug resistance was observed.

**Conclusion:**

Several pathogens were resistant to greater than five antimicrobial agents, so that proper management of patients with bacteremia is needed, and a careful selection of effective antibiotics should be practiced.

## 1. Background

Bacterial bloodstream infections constitute a significant public health problem and present an important cause of morbidity and mortality in HIV-infected patients. Even if it is worldwide, the highest burden is documented in sub-Saharan countries including Ethiopia [1]. Bacteremia is one of the major leading causes of deaths in HIV-infected patients as compared to without HIV [2]. Conditions such as defective cell-mediated immunity, altered B cell function, and defect of neutrophil are leading factors that enhance susceptibility of patients to bacterial infections [3]. Sepsis is another episode of disease among HIV/AIDS and remains the second leading cause of death with an estimated mortality rate of 7% in the pediatric population [2]. Most of these deaths were reported from facilities in sub-Saharan Africa and Asia where healthcare access, infrastructure, and staffing remains suboptimal. Both Gram-negative and Gram-positive bacterial agents can play a pivotal role in causing high morbidity of blood stream infections. For instance, a survey among HIV-I patients in Malawi showed that 30% had bloodstream infections, and organisms isolated in this study were mainly *S. pneumoniae*, *S. aureus, E. coli, K. pneumoniae*, and *M. tuberculosis* [2].

Antimicrobial resistance (AMR) is an emerging and serious public health problem among HIV/AIDS patients in both developed and developing countries, though the burden is high in sub-Saharan countries [4–6]. Different studies show that antimicrobial resistance is alarmingly increasing. Mutation and selective pressure by antibiotics during treatment are among documented factors for antimicrobial resistance [7, 8]. Rising antibiotic resistance rates among *E. coli* (with resistance to third generation cephalosporins and fluoroquinolones) is particularly problematic, since cephalosporins are the mainstay of empiric therapy for both community-acquired and hospital-acquired bloodstream infections in resource-limited settings. The estimated prevalence of extended spectrum beta-lactamase- (ESBL-) producing Enterobacteriaceae in Asia and sub-Saharan Africa is between 60% and 90% [9], highlighting the growing challenge of treating bloodstream infections in these countries [10]. In developing countries, controlling AMR is still challenging because of lack of surveillance systems, poor infection control measures, and self-prescription of antimicrobial agents [11]. An infection with multidrug-resistant bacteria also consumes more healthcare resources [11, 12]. Therefore, this study aimed to assess the antimicrobial resistance patterns of bacterial isolates from blood culture among HIV/AIDS patients at Felege Hiwot Referral Hospital and to provide updated information to regulatory bodies and those who would like to use the findings for the development of intervention strategies as appropriate.

## 2. Materials and Methods

### 2.1. Study Setting, Design, Population, and Sampling Techniques

An institution-based cross-sectional study was conducted at Felege Hiwot Comprehensive Specialized Hospital, Bahir Dar, Amhara regional state. It was performed to determine antimicrobial resistance patterns of bacterial isolates among bloodstream-suspected HIV/AIDS patients attending ART clinic from July 11 to December 11, 2018. Bahir Dar city is located 576 km from the capital city of the country, Addis Ababa. Based on the 2007 Census conducted by the Central Statistical Agency (Ethiopia), this city has a total population of 221,990 [13]. The hospital was purposely selected because it is one of the biggest referral hospitals in the region. Currently, it provides inpatient and outpatient services for more than 7 million people in its catchment area and nearby regions. The hospital has inpatient wards, outpatient department, and laboratory service with a total of 400 beds and 9 operating tables. Besides, it has an ART clinic with separate ART Laboratory and ART Pharmacy that serve about 17,569 HIV patients [14]. The sample size (384) was determined by using a single population proportion formula while considering the prevalence of 50%, with a 95% confidence interval, and a 5% margin of error. Study participants were recruited by the convenient sampling technique. HIV/AIDS patients suspected of bloodstream infection were included in this study. However, patients who took antibiotics two weeks before data collection and at the time of the study were excluded. In this study, all patients visiting Felege Hiwot Comprehensive and Specialized Hospital for a certain clinical disease diagnosis were considered as a source population. Whereas HIV/AIDS patients who were visiting the ART clinic for bloodstream infection diagnosis during the study period were considered as a target population.

### 2.2. Data Collection Methods

A pretested, structured questionnaire was prepared from reviewed literature and used to collect sociodemographic information. It was prepared in English, translated to the local language (Amharic), and then translated back into English to check its consistency. Clinical signs such as axillary temperature ≥38.5°C or ≤36.5°C, pulse ≥90°beats/minute, and frequency of respiration ≥20 minute were used as criteria to suspect bloodstream infection and recruit patients for this study.

### 2.3. Laboratory Methods

#### 2.3.1. Blood Sample Collection

Venous blood samples, 20 mL (adult) and 2–5 ml (children), were taken from each patient, and 10 mL (adult) and 2–5 mL (children) were inoculated into a pair of bottles (Becton, Dickinson, USA) under strict aseptic procedures [12].

#### 2.3.2. Culture and Identification

The blood samples were subsequently incubated for 7 days according to the standards of the World Health Organization (WHO) [15]. The presence of bacteria was indicated when there was evidence of visible microbial growth, hemolysis, turbidity, gas production, and/or coagulation of the broth. About 0.1 ml of the sample was drawn using a sterile syringe from culture bottles showing at least one of the abovementioned indications and plated out on MacConkey, blood, and chocolate agar plates using the streak plate technique. The plates were then incubated at 37°C for 18–24 hours and observed for bacterial growth. The chocolate agar plates were incubated under microaerophilic conditions in a jar for the possible isolation of microaerophiles. Colonies appearing on the agar plates were subcultured for purity, and a minimum of three colonies with identical morphology was selected individually and subjected to identification by standard biochemical tests. Gram-negative bacteria were identified by the oxidase test, indole production, citrate utilization, motility, urease, oxidase, gas production, hydrogen sulfide production, carbohydrate fermentation, and lysine decarboxylases production tests [12]. Similarly, Gram-positive bacterial isolates were mainly identified using coagulase and catalase tests. Further differentiation of *Streptococcus* species was performed by bacitracin/optochin tests. The identification of *Haemophilus* species was performed using the XV factor-supplemented chocolate agar plate and blood agar plate. Samples were recorded as negative when no growth was detected within seven days' incubation [12].

#### 2.3.3. Antimicrobial Susceptibility Test

Antimicrobial susceptibility tests were carried out using the Kirby–Bauer disc diffusion method as per the Clinical Laboratory Standards Institute (CLSI) guidelines on Muller–Hinton agar [16]. The suspension of 3–5 pure colonies of freshly grown test organisms was prepared equivalent to 0.5 McFarland standards. The surface of Muller–Hinton agar was then completely covered by rotating the swab with the suspension. For fastidious microorganisms, Muller–Hinton agar supplemented with 5% lysed/defibrinated whole blood was used [17]. The plates were allowed to dry for 3–5 minutes; then, discs were evenly distributed with 24 mm apart on the inoculated plate using sterile forceps and incubated at 37°C for 18–24 hours. The diameter of the zone of inhibition around the disc was measured using a ruler. Results were interpreted as sensitive, intermediate, and resistance based on the CLSI 2016 guideline [16].

The following routinely used antimicrobials were tested: ampicillin (10 *μ*g), amoxicillin–clavulanic acid (30 *μ*g), ceftriaxone (30 *μ*g), ciprofloxacin (5 *μ*g), chloramphenicol (30 *μ*g), sulfamethoxazole/trimethoprim (25 *μ*g), gentamicin (10 *μ*g), piperacillin (100 *μ*g), tetracycline (30 *μ*g), penicillin (10 IU), vancomycin (30 *μ*g), erythromycin (15 *μ*g), oxacillin (1 *μ*g), clindamycin (2 *μ*g), and cefoxitin (30 *μ*g). Cefoxitin disc (30 *μ*g) was used for the detection of MRSA and MR–CoNS. Culture media were incubated at 37°C for 16 to 18 hours for MRSA and 24 hours for coagulase-negative *Staphylococci* (CoNS). Sensitivity results for MRSA and MR–CoNS were reported when the zone of diameter is ≤21 mm and ≤24 mm, respectively [16]. Multidrug resistance was defined as the resistance of an isolate to three or more antimicrobial classes tested [18].

#### 2.3.4. Quality Control

Standard reference strains of *E. coli* (ATCC–25922), *S. aureus* (ATCC–25923), *P. aeruginosa* (ATCC–27853), *E. faecalis* (ATCC 29212), *S*. *pneumoniae* (ATCC 49619), and *S. pyogenes* (ATCC 19615) were used as a media quality control throughout the study.

### 2.4. Data Analysis and Interpretation

Collected data were entered into Epi-data 3.1 and exported to the SPSS version 20 statistical software for analysis. During analysis, descriptive statistics including mean, frequency, and percentage were used to summarize the data as appropriate. Then, the findings of this study were presented in the form of texts, tables, and graphs as appropriate. A *P* value of <0.05 was considered as statistically significant.

### 2.5. Ethical Consideration

An official ethical letter (protocol number: DRERC/182/15/MLS) was obtained from the Departmental Research and Ethics Review Committee of Addis Ababa University, Department of Medical Laboratory Sciences. Then, consent was obtained from the study participants after informing their involvement was based on a voluntary basis. Participants who were unwilling to involve in the study and those who need to quit their participation at any stage were informed to do so without any restriction.

## 3. Results

### 3.1. Demographic Characteristics

A total of 384 bacteremia suspected patients were included in this study. Of these, 157 (41%) were males and 227 (59%) were females. They were aged 1–75 years with a mean age of 36 years (SD ± 11.8). Distribution according to the age range showed that the majority of the HIV-infected patients were in the age group of 25–44 years, 252 (65.6%), followed by those whose age ranged between 45 years and 64 years, 83 (21.8%), and the least number was between the age group of 15 years and 24 years, 20 (5.3%) (Table 1). About 123 (32%) samples were culture-positive, with all of them showing a monobacterial growth, yielding a total of 123 bacterial isolates. A higher positivity rate was seen in children as compared to the adults (Table 1).

### 3.2. Bacterial Isolates

**Figure 1:**
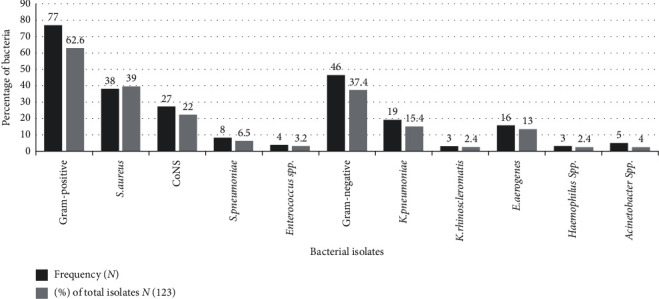
Distribution of bacterial isolates among blood culture-positive HIV/AIDS patient at Felege Hiwot Referral Hospital.

The bacterial profile from the blood culture of HIV-infected patients revealed that Gram-positive bacterial isolates had the highest percentage of 62.6% followed by Gram-negatives with 37.4%. Among Gram-positives, *S. aureus,* 49.4% (38/77), was the most predominant followed by CoNS, 35% (27/77), whereas *K. pneumoniae* was the leading pathogen, 41.3% (19/46), among Gram-negatives (Figure 1).

### 3.3. Antibiotic Susceptibility Profile of Gram-Positive Bacterial Isolates

*S. aureus* isolates exhibited the highest percentage resistance to penicillin, tetracycline, and cotrimoxazole with 36 (95%), 27 (71%), and 25 (65%), respectively (Table 2). Besides, 28 (73.7%) *S. aureus* and 18 (70%) CoNS were methicillin-resistant (Table 3). Among the total number of MRSA isolates, 25% (7/28) were healthcare-associated, while 21/28 (75%) were community-associated. About 11/18 (61.1%) of MR-CoNS isolates were healthcare associated and 7/18 (38.9%) were community associated (Table 3).

### 3.4. Antibiotic Susceptibility Profile of Gram-Negative Bacterial Isolates

*K. pneumoniae* showed a significant level of resistance to tetracycline, 18 (95%), and chloramphenicol, 17 (89%). *E. aerogenes* also showed the highest percentage of resistance to ampicillin, 15 (93.7%), and tetracycline, 13 (81.3%) (Table 4).

Regarding multidrug-resistance patterns of bacterial isolates, about 78% (96/123) of them showed multidrug resistant (MDR). Even about 50 (40.7%) of the isolates were resistant to at least five classes of antimicrobial agents (Table 5). The most common MDR Gram-positive bacterial isolates were *S. aureus,* 48% (37/77), CoNS, 28.6% (22/77), and *K. pneumoniae*, 23.9% (11/46) (Table 5).

## 4. Discussion

Bloodstream bacterial infection is one of the most common infections among HIV-infected individuals [19]. In this study, the overall prevalence of bloodstream infections among HIV/AIDS patients attending the Felege Hiwot Referral Hospital was 32%. This prevalence is higher than the findings reported in Nigeria, which was 22.9% [3]. However, our result is comparable with the results reported at Gondar Teaching Referral Hospital, which was 31% [17]. On the contrary, our result is much lower than the findings reported in China [20] and the USA [21], which is 40% and 80%, respectively.

The antibiotic susceptibility test results on the current study showed that 40.7% of the bacterial isolates were resistant to more than five antimicrobial agents. This percentage was significantly higher than the previous finding documented in Gondar, Ethiopia, and 29% of bacterial isolates were resistant to greater than five antimicrobial agents [17]. The reason for the higher resistance in the current study might be due to the inappropriate use of broad-spectrum antibiotics. This study also illustrated that 58.4% of Gram-positive and 43.4% of Gram-negative bacterial isolates were resistant to cotrimoxazole [17]. Although it is very difficult to generalize with this sample size, this study showed the highest resistant profile of many bacterial isolates to cotrimoxazole. Therefore, a large-scale comparative study in the future will answer this critical question. However, this percentage was much lower than results reported in Gondar, Ethiopia, in which 65.3% of the Gram-positive and 100% of the Gram-negative bacterial isolates were resistant to cotrimoxazole [17]. Another study from Tanzania also showed that 81.3% Gram-positive and 75% of Gram-negative bacterial isolates were resistant to cotrimoxazole [22].

In the current study, more than 93% of Gram-positive and Gram-negative bacterial isolates showed higher sensitivity to ciprofloxacin. More interestingly, all *Enterococcus* species and *S. pneumoniae* species were 100% sensitive to vancomycin (Table 2). On another hand, 98% and 93.5% of Gram-negative bacteria showed good sensitivity to ceftriaxone and chloramphenicol, respectively (Table 3). This percentage is slightly comparable to a study conducted in Gondar town, Ethiopia, in which 100% of Gram-negative and greater than 75% of Gram-positive bacteria were sensitive to ciprofloxacin [17]. Similarly, the study in Kampala, Uganda, among HIV-1 infected children showed that more than 80% of the bacterial isolates were susceptible to ciprofloxacin [23]. Another study from India indicated that among bacterial isolates, ceftriaxone and ciprofloxacin were effective with a very low resistance of 8% and 30.3%, respectively [24].

In this study, a significant level of resistance to methicillin by S*. aureus* and coagulase-negative *Staphylococci* (CoNS) was observed, yielding 73.7% and 66.7% of methicillin-resistant *S. aureus* (MRSA) and methicillin-resistant coagulase-negative *Staphylococci* (MR-CoNS). This percentage of MRSA was significantly higher than findings reported in Bahir Dar, Ethiopia [25], and Italy, in which about 16.8% and 32%, respectively, were MRSA among HIV/AIDS patients [26].

## 5. Limitations

This study did not consider the antimicrobial resistance profile of some bacterial isolates such as *Mycobacterium* and anaerobic bacteria due to resource constraints, and also, it did not include parameters such as mortality rate and predisposing factors.

## 6. Conclusions

Based on our findings, we can conclude that the antimicrobial resistance patterns were high. Ciprofloxacin and vancomycin were found to be an active antibacterial agent for Gram-positive bacteria, while ceftriaxone, ciprofloxacin, and chloramphenicol were effective for Gram-negative bacteria. Most of the bacterial isolates in this study, 78% of them, showed MDR including cotrimoxazole, which is the most commonly prescribed prophylaxis agent in ART clinics. Blood culture tests should be considered for better management of bacterial bloodstream infections among HIV/AIDS patients. We recommend an additional study regarding mortality rate, loss of cost, and time due to bloodstream infection among HIV/AIDs patients. Besides, there should be continuous antimicrobial resistance surveillance in the study area on a large scale.

## Figures and Tables

**Table 1:** Demographical characteristics of study participants with bacterial bloodstream infection among HIV/AIDS patients in Felege Hiwot Referral Hospital.

Characteristic	Bacterial status		Total, *N* (%)
	Positive, *N* (%)	Negative, *N* (%)	
Sex			
Male	50 (31.8)	107 (68)	157 (41)
Female	73 (32.1)	154 (67.8)	227 (59)
Age			
1–14	14 (53.8)	12 (46.2)	26 (6.8)
15–24	5 (25)	15 (75)	20 (5.3)
25–44	81 (32.1)	171 (67.8)	252 (65.6)
45–64	23 (27.7)	60 (72.3)	83 (21.8)
>65	0 (0)	3 (100)	3 (0.8)
Marital status			
Unmarried	20 (36.2)	45 (63.8)	65 (24.5)
Married	57 (29.8)	135 (70.2)	191 (49.7)
Widow	3 (27.3)	8 (72.7)	11 (2.9)
Divorced	73 (33)	43 (67)	88 (22.9)
Educational level			
Illiterate	39 (30.7)	72 (69.3)	104 (27.3)
Literate	90 (32.6)	186 (67.4)	276 (72.7)
Occupational			
Farmer	11 (29.7)	26 (70.3)	37 (9.6)
Student	16 (32)	34 (68)	50 (13)
House wife	20 (39.2)	32 (61.5)	51 (13.2)
Employer	61 (30.6)	156 (71.9)	236 (61.5)
Other	15 (53.6)	13 (46.4)	10 (2.6)
Total	123 (32)	261 (68)	384 (100)

*Note.* A, government and self-employed; b, jobless and children under 4 years. Age is categorized based on the WHO guidelines.

**Table 2:** Antibiotic resistance patterns of Gram-positive bacterial isolates among HIV/AIDS-infected patients at Felege Hiwot Referral Hospital.

Gram-positive	Antibiotic resistance profile									
	CIP,	COT,	CLI,	E,	OXA,	FOX,	VA,	TE,	*P,*	*C*,
	*N* (%)	*N* (%)	*N* (%)	*N* (%)	*N* (%)	*N* (%)	*N* (%)	*N* (%)	*N* (%)	*N* (%)
*S. aureus* (38)										
*S*	36 (95)	12 (32)	27 (71)	12 (31.5)	9 (24)	10 (26.3)	—	9 (23.6)	2 (5.2)	—
*I*	1 (2.5)	1 (2.6)	0 (0)	4 (10.5)	—	—	—	2 (5.2)	—	—
*R*	1 (2.5)	25 (65)	11 (29)	22 (58)	28 (73.6)	28 (73.7)	—	27 (71)	36 (95)	—
CoNS (27)										
*S*	25 (92.5)	11 (41)	19 (70)	10 (37)	8 (30)	9 (33.3)	—	7 (26)	2 (7.4)	—
*I*	0 (0)	2 (7.4)	0 (0)	3 (11)	—	—	—	1 (3.7)	—	—
*R*	2 (7.5)	14 (52)	8 (30)	14 (52)	19 (70)	18 (66.7)	—	19 (70.3)	25 (93)	—
*Enterococcus* spp. (4)										
*S*	4 (100)	2 (50)	—	3 (75)	—	—	4 (100)	0 (0)	0 (0)	4 (100)
*I*	0 (0)	0 (0)	—	0 (0)	—	—	0 (0)	1 (25)	0 (0)	0 (0)
*R*	0 (0)	2 (50)	—	1 (25)	—	—	0 (0)	3 (75)	4 (100)	0 (0)
*S. pneumoniae* (8)										
*S*	7 (87.5)	4 (50)	—	5 (62.5)	—	—	8 (100)	2 (25)	1 (12.5)	5 (62.5)
*I*	0 (0)	0 (0)	—	0 (0)	—	—	0 (0)	0 (0)	0 (0)	3 (37.5)
*R*	1 (12.5)	4 (50)	—	3 (37.5)	—	—	0 (0)	6 (75)	7 (87.5)	0 (0)
Total (*n* = 77)										
*S*	72 (93.5)	29 (37.6)	—	30 (39)	—	—	69 (90)	18 (23.4)	5 (6.5)	—
*I*	1 (1.3)	3 (3.9)	—	7 (9)	—	—	2 (2.6)	4 (5.2)	0 (0)	—
*R*	4 (5.2)	45 (58.4)	—	40 (52)	—	—	6 (7.7)	55 (71.4)	72 (95.5)	—

*Note.* CIP, ciprofloxacin; COT, cotrimoxazole; *E*, erythromycin; *P,* penicillin; VA, vancomycin; OXA, oxacillin; CLI, clindamycin; FOX, cefoxitin; CN, gentamycin; TE, tetracycline; *S,* sensitivity, I, intermediate; *R,* resistance; CONS, coagulase-negative *Staphylococci*.

**Table 3:** Methicillin-resistant *Staphylococci* among HIV/AIDS patients at Felege Hiwot Referral Hospital.

MR *Staphylococci*	Place of acquisition	
	HCA BSIs, *N* (%)	CA BSIs, *N* (%)
MRSA (*n* = 28)	7 (25)	21 (75)
MR-CoNS (*n* = 18)	11 (61.1)	7 (38.9)
Total (*n* = 46)	18 (39.1)	27 (59.8)

*Note.* MRSA, methicillin-resistant *S. aureus*, MR-CoNS, methicillin-resistant coagulase-negative *Staphylococci*, HCA BSIs, healthcare-associated bloodstream infections, CA BSIs, community-associated bloodstream infections.

**Table 4:** Antibiotic resistance pattern of Gram-negative bacterial isolates among HIV/AIDS patients at Felege Hiwot Referral Hospital.

Gram-negative	Antibiotic susceptibility pattern								
	CIP,	COT,	AMP,	AMC,	CRO,	CN,	TE,	C,	PI,
	*N* (%)	*N* (%)	*N* (%)	*N* (%)	*N* (%)	*N* (%)	*N* (%)	*N* (%)	*N* (%)
*K. pneumoniae* (*n* = 19)									
* S*	19 (100)	12 (63)	2 (10.5)	14 (73.6)	19 (100)	8 (42)	1 (5)	16 (84.2)	—
* I*	0 (0)	0 (0)	0 (0)	1 (5.3)	0 (0)	1 (5.3)	0 (0)	0 (0)	—
* R*	0 (0)	7 (37)	17 (89)	4 (21)	0 (0)	10 (53)	18 (95)	3 (16)	—
*K. rhinoscleromatis* (*n* = 3)									
* S*	2 (67)	2 (67)	1 (33.3)	2 (66.7)	3 (100)	3 (100)	0 (0)	3 (100)	—
* R*	1 (33)	1 (33)	2 (66.7)	1 (33.3)	0 (0)	0 (0)	3 (100)	0 (0)	—
*E. aerogenes* (*n* = 16)									
* S*	14 (87.5)	6 (37.5)	1 (6.3)	10 (62.5)	15 (93.7)	6 (37.5)	1 (6.25)	16 (100)	—
* I*	0 (0)	0 (0)	0 (0)	0 (0)	0 (0)	1 (6.2)	2 (12.5)	0 (0)	—
* R*	2 (12.5)	10 (62.5)	15 (93.7)	6 (37.5)	1 (6.3)	9 (56.2)	13 (81.3)	0 (0)	—
*Haemophilus* spp. (*n* = 3)									
* S*	3 (100)	1 (33.3)	0 (0)	2 (66.7)	3 (100)	2 (66.7)	0 (0)	3 (100)	—
* R*	0 (0)	2 (66.7)	3 (100)	1 (33.3)	0 (0)	1 (33.3)	3 (100)	0 (0)	—
*Acinetobacter* spp. (*n* = 5)									
* S*	5 (100)	5 (100)	0 (0)	3 (60)	5 (100)	3 (60)	0 (0)	5 (100)	3 (60)
* R*	0 (0)	0 (0)	5 (100)	2 (40)	0 (0)	2 (40)	5 (100)	0 (0)	2 (40)
Total (*N* = 46)									
* S*	43 (93.5)	26 (56.5)	4 (8.7)	31 (67.4)	45 (98)	22 (47.8)	2 (4.3)	43 (93.5)	—
* I*	0 (0)	0 (0)	0 (0)	1 (2.1)	0 (0)	2 (4.3)	2 (4.3)	0 (0)	—
* R*	3 (6.5)	20 (43.5)	42 (91.3)	14 (30.4)	1 ((2.2)	22 (47.8)	42 (91.3)	3 (6.5)	—

*Note.* CIP, ciprofloxacin; COT, cotrimoxazole; CN, gentamicin; AMP, ampicillin; AMC, amoxicillin–clavulanic acid; TE, tetracycline; CRO. ceftriaxone; *C,* chloramphenicol; Pi, piperacillin; *S,* sensitivity; *I*, intermediate; *R,* resistance.

**Table 5:** Multidrug-resistant patters of the bacterial isolates among blood culture-positive HIV/AIDS patients at Felege Hiwot Referral Hospital.

Bacterial isolate	Class of drugs						
	R0, *N* (%)	R1, *N* (%)	R2, *N* (%)	R3, *N* (%)	R4, *N* (%)	≥R5 (%)	Overall MDR (%)
Gram-positive							
*S. aureus* (*n* = 38)	0 (0)	1 (2.6)	0 (0)	7 (18.4)	4 (10.5)	26 (68.4)	37 (48)
CoNS (*n* = 27)	0 (0)	2 (7.4)	3 (11.1)	4 (14.8)	5 (18.5)	13 (48.1)	22 (28.6)
*S pneumoniae* (*n* = 8)	0 (0)	1 (12.5)	1 (12.5)	4 (50)	0 (0)	2 (25)	6 (7.8)
*Enterococcus* spp. (*n* = 4)	0 (0)	1 (25)	0 (0)	1 (25)	1 (25)	1 (25)	3 (3.9)
Total (*n* = 77)	0 (0)	5 (6.5)	4 (5.2)	16 (20.8)	10 (13)	42 (54.5)	68 (88.3)
Gram-negative							
*K pneumoniae* (*n* = 19)	2 (10.5)	0 (0)	6 (31.5)	4 (21)	2 (10.5)	5 (26.3)	11 (23.9)
*K. rhinoscleromatis* (*n* = 3)	0 (0)	0 (0)	0 (0)	3 (100)	0 (0)	0 (0)	3 (6.5)
*E aerogenes* (*n* = 16)	0 (0)	0 (0)	3 (18.8)	7 (43.7)	3 (18.8)	3 (18.8)	3 (6.5)
*Haemophilus* spp. (*n* = 3)	0 (0)	1 (33.3)	2 (66.7)	0 (0)	0 (0)	0 (0)	0 (0)
*Acinetobacter* spp. (*n* = 5)	0 (0)	3 (60)	1 (20)	1 (20)	0 (0)	0 (0)	1 (2.2)
Total (*n* = 46)	2 (4.3)	4 (8.7)	12 (28.2)	15 (32.6)	5 (11)	8 (17.3)	28 (60.8)
Overall (*n* = 123)	2 (1.6)	9 (7.3)	16 (13)	31 (25.2)	15 (12.1)	50 (40.7)	96 (78)

*Note. R*_0_, nonresistance; *R*_1_, resistance for 1 antibiotic; *R*_2_, resistance for 2 antibiotic; *R*_3_, resistance for 3 antibiotics; *R*_4_, resistance for 4 antibiotic; *R*_5_, resistance for 5 and above.

## Data Availability

The data used to support this study are available from the corresponding author upon request.

## Abbreviations

AIDS: Acquired immunodeficiency syndrome
ART: Antiretroviral therapy
AMR: Antimicrobial resistance
BAP: Blood agar plate
CAP: Chocolate agar plate
CLSI: Clinical and Laboratory Standards Institute
CONS: Coagulase-negative *Staphylococci*
ECDC: European Centre for Disease Prevention and Control
HIV: Human immunodeficiency virus
MR-CoNS: Methicillin-resistant coagulase-negative Staphylococci
MRSA: Methicillin-resistant *Staphylococcus aureus*.
